# Performance of elite maize genotypes under selected sustainable intensification options in Kenya

**DOI:** 10.1016/j.fcr.2020.107738

**Published:** 2020-04-01

**Authors:** Leonard Rusinamhodzi, Dan Makumbi, James M. Njeru, Fred Kanampiu

**Affiliations:** a International Institute of Tropical Agriculture (IITA), CSIR Campus, PMB 56, Legon, Accra Ghana; b International Maize and Wheat Improvement Center (CIMMYT), ICRAF Campus, P.O Box 1041-00621, Nairobi, Kenya; c International Institute of Tropical Agriculture (IITA), ICIPE Campus Duduville, P.O. Box 30772-00100, Nairobi, Kenya

**Keywords:** Conservation agriculture, Conventional tillage, Maize-Cowpea intercropping, Long-Term experiment, Crop competition, Sustainability, genotype stability

## Abstract

Combining different cropping and tillage systems with different genotypes across several cropping seasons can reveal opportunities for sustainable intensification (SI). The objective of this study was to assess the performance of six maize genotypes under intercropping with conservation tillage (no-till) – two promising options for SI. The experiment was carried out over three years (or six cropping seasons) at Kiboko Research Station, Kenya with sole cropping and mouldboard ploughing as baseline production systems. Results showed that maize genotypes and cropping systems had a significant effect on yield, but the effect of tillage was not significant. Moreover, there was no significant interactive effects of the tested factors on maize yield. The maize genotype CKH10085 had the highest yield of 7.7 t ha^−1^ under sole cropping yet it also recorded the largest yield penalty due to intercropping of 1.1 t ha^−1^. On the other hand, genotype CKH10717 maintained the same average yield of 7.1 t ha^−1^ in both conventional and conservation tillage systems. The commercial genotype genotype CKH10080 and CKH08051 were more stable than the other experimental genotypes under the variable growing and management conditions. These two genotypes are of intermediate maturity and drought tolerance, two critical attributes to improved maize production. Intercropping reduced maize yields due to increased competition, for example the overall yield of sole cropping was 7.1 t ha^-1^ compared with 6.4 t ha^-1^ under intercropping; representing an overall yield penalty of 0.7 t ha^-1^. The differences in performance of maize genotypes revealed opportunities to deploy genotypes to reduce risk or maximize yield, depending on the biophysical circumstances and the production objective of the farmer.

## Highlights

• We evaluated 6 maize genotypes under two tillage and two cropping systems.
• CA plots resulted in marginally lower maize yields than conventional tillage system.
• Intercropping reduced maize yields due to increased competition.
• Genotypes can be deployed in response to need for risk reduction or yield increase.
• Targeting of CA and reducing interspecific competition are key necessities.

## 1 Introduction

The farming systems in Kenya are diverse but largely dominated by maize (*Zea mays* L.) production because maize is an important staple food and cash crop for a large proportion of the population. Maize accounts for more than 40 % of total staple food caloric intake (Muhunyu, 2009). Despite this importance and the efforts farmers invest in production, yields have not increased due to a plethora of challenges including poor and declining soil fertility, erratic rainfall and generally the poor resource endowments that limit the options and opportunities for farmers to address site specific production constraints (e.g. Sanchez, 2002; Sanginga and Woomer, 2009). Recent estimates show the average maize yield in Kenya to be 1.6 t ha^−1^ compared with a regional (East Africa) average of 2.5 t ha^−1^ (Adhikari et al., 2015). Crop diversification and improved management are urgently needed for smallholder farmers in this region to reduce the risk of crop failure (e.g. Challinor et al., 2007). Increased crop diversity besides ensuring improved nutrition also reduces the impact of pests and diseases outbreaks by providing more habitats for predatory insects (Trenbath, 1993). Maize-legume intercropping remains the most common and widely practiced form of crop diversification in East Africa (Giller, 2001; Mucheru-Muna et al., 2009). However, intercropping needs adjustments with respect to planting, cultivation, fertilization, pest-control and harvesting of more than one crop in the same field (Machado, 2009). The challenge here is how to design improved crop production systems that are appropriate for the prevailing farming systems without creating new constraints for resource-poor farmers.

Sustainable intensification (SI) is a process where crop yields can be increased through increased resource use and resource use efficiency, without land expansion and with minimal adverse environmental impact (cf. Doré et al., 2011; Pretty et al., 2011; Bommarco et al., 2013; Struik and Kuyper, 2017). This approach is currently the basis upon which the prospects of feeding more than 9 billion people in ca. 2050 while improving the environment are premised (Tilman et al., 2011). According to Spiertz (2013), agronomists and plant breeders can jointly improve crop performance by introducing new technologies and farming practices, and by exploiting new knowledge on genetic traits and physiological relationships in advanced breeding programs for genotypes that are tolerant to multiple stresses such as drought, heat and salinity. A better quantitative understanding of Genotype × Environment × Management (G × E × M) interactions is needed to achieve SI. The International Maize and Wheat Improvement Center (CIMMYT) with partners have developed improved maize genotypes tolerant to multiple stresses such as limited moisture and low nitrogen (N). However, it is not known how these attributes are expressed when grown under different cropping systems used by smallholder farmers.

Several SI options have been suggested within the broad framework of integrated soil fertility management (ISFM) for smallholder farms in Africa (Vanlauwe et al., 2010), and these include agroforestry, grain legumes, green manures, germplasm, inorganic fertilizers, cattle manure, local adaptation, and conservation agriculture (CA). Conservation agriculture refers to a farming system that maintains permanent soil cover through previous crop residues, minimum soil disturbance and crop diversification in space and time (FAO, 2008). CA increases infiltration and reduces soil erosion and thus can be important in situations of erratic rainfall distribution and seasonal dry spells where higher moisture conservation during critical crop phases may increase crop yields or reduce the risk of crop failure (Thierfelder and Wall, 2010; Rusinamhodzi et al., 2012). CA may also improve soil fertility following the build-up of carbon in the soil and reduce costs related to land preparation and weeding (Thierfelder et al., 2014), though results vary from place to place (Giller et al., 2009; Rusinamhodzi et al., 2011).

In the past decade, considerable efforts have been invested in research and out-scaling of CA as the most suitable SI option that farmers could utilize. Initiatives based on integrating legumes into the maize-based farming systems have shown promise due to the dual role of legumes in improving diet and soil fertility as well as their general low cost and local availability (e.g. Giller, 2001; Mafongoya et al., 2006). In the current study, we assessed evidence of SI by analyzing maize productivity from a long-term experiment consisting of six maize genotypes, two cropping systems (sole vs. intercropping with cowpea) and two tillage systems (conventional vs. conservation) over six cropping seasons. The study site is predominantly water-limited, and to reduce environmental variability and total crop failure, we applied supplemental irrigation uniformly across all plots. The underlying hypothesis is that improved understanding of the interactions among genotype, and management decisions (*G* × *M)*, and the associated outcomes will facilitate the deployment of crop genotypes and the necessary production systems in target environments where they are most suited.

## 2 Materials and methods

### 2.1 Site description

The experiment was carried out at Kenya Agricultural and Livestock Research Organization’s Kiboko Crops Research Station in Kenya (37.7235 °E, 2.2172 °S, 975 m above sea level) from the short rainy season (September) of 2013 until the long rainy season (March) of 2016 representing six cropping seasons. Kiboko receives between 545 and 629 mm of rainfall per year distributed over two seasons i.e. the long rainy season (LR) season (March to July) while the short rain (SR) season (October and January). Thus, success of maize production depends heavily on supplemental irrigation during the whole growing season at this research station. The climate is generally classified as hot and dry with mean annual maximum temperature of 28.6 °C and mean annual minimum of 15.5 °C (CIMMYT, 2013). The soils at Kiboko are well drained, very deep dark reddish brown to dark red, friable sandy clay to clay classified as Acri-Rhodic Ferrassols. These soils have been developed from undifferentiated basement system rocks, mostly banded gneisses (CIMMYT, 2013).

### 2.2 Description of experiments

The experimental design was a randomized complete block design with three replications and laid out in split-split-plot arrangement. The main plots were assigned to tillage systems (conservation agriculture vs. conventional tillage), the sub-plots were assigned to cropping systems (sole cropping vs. intercropping) while the sub-sub plots were assigned to six maize genotypes. At planting, a minimum of 2.5 t ha^−1^ crop residue cover was maintained in the plots during the initial season, and in subsequent seasons all crop residues in conservation tillage (no-till) plots were retained *in situ*. Conventional tillage was characterized by soil inversion through tillage and removal of crop residues. We did not have sole cowpea treatment, in line with local production systems where legumes are often intercropped with maize. Due to this limitation with the design (i.e. absence of sole cowpea plot) we did not focus cowpea productivity and only focus was on how intercropping and tillage would affect the performance of the superior genotypes. The maize genotypes used in this study were experimental genotypes CKH08051, CKH10077, CKH10080, CKH10085, and CKH10717 developed by CIMMYT under the Drought Tolerant Maize for Africa (DTMA) project, and commercial genotype H513. The experimental maize genotypes had been tested extensively in regional trials at 26 locations in eastern Africa and showed good yield potential under a range of growing conditions and resistance to major foliar diseases such as gray leaf spot (caused by *Cercospora zeae-maydis* Tehon & E. Y. Daniels), northern corn leaf blight (caused by *Exserohilum turcicum* (Pass.) K. J. Leonard & Suggs), and maize streak virus (Makumbi, 2011). Experimental genotypes CKH08051, CKH10077, CKH10080 are of intermediate maturity (500 series according to FAO classification) and drought tolerant. Genotype CKH10717 is drought tolerant, of intermediate to late maturity, and is marketed as IFFA630 (also locally named ‘Lubango’) and widely grown in the northern and lake zones of Tanzania. Genotype CKH10085 is drought tolerant, of intermediate maturity and will be available as a commercial genotype in Kenya in 2020. Genotype H513 is a popular maize genotype grown widely in the mid-altitude areas (1000-1500 masl) of Kenya. The combination of maize genotypes and tillage and cropping systems gave a total of 24 treatments that were replicated three times to give a total of 72 plots.

The plot sizes measured 7 m wide × 6 m long. Maize was planted at a spacing of 0.75 m  ×  0.25 m with one plant per hill to give a total population density of approximately 53, 333 plants per hectare. In the intercrop, cowpea was sown between the maize rows on the same day with a row maize alternating with a row of cowpea. The in-row spacing for cowpea was 0.2 m in additive design to give a total plant population in the intercrop of approximately 120, 000 plants per hectare. The cowpea genotype was the bushy and semi-spreading genotype commercially known as M66, suited for arid and semi-arid areas and also tolerant to cowpea yellow mosaic virus.

All plots received 100 kg ha^−1^ of DAP (18 %N: 46 %P_2_O_5_: 0 %K) at planting on the maize crop only. A top dressing of 80 kg N ha^−1^ in the form of urea was applied on the maize crop four weeks after planting. The plots were kept weed free by using the hand hoe for weeding in the conventional tillage plots. In no-till plots, glyphosate (N- (phosphono-methyl) glycine) was used at a rate of 3.5 l ha^−1^ before planting for weed control. No-till plots were kept weed free by scratching on the soil surface with a hand hoe. Supplemental irrigation was used due to limited rainfall received across the years and cropping seasons. Rainfall data was captured from a meteorological station located at the experimental site at Kiboko. The total rainfall received for the six cropping seasons was 174 mm (2013 SR), 184 mm (2014 LR), 147 mm (2014 SR), 218 mm (2015 LR), 110 mm (2015 SR), and 92 mm (2016 LR). Supplemental water was only added when there was no rainfall to avoid water stress during crop growth, but planting was done when natural rainfall was sufficient to initiate germination. Irrigation was initiated after a rain free period of at least three days and based on the visual appearance of plants. Irrigation water was applied uniformly across treatments for approximately three hours at a rate of approximately 10 liters per hour via drip irrigation. Supplemental irrigation was important to reduce environmental variability and total crop failure, allowing the assessment of technical solutions such as elite genotypes, cropping and tillage systems developed for rainfed conditions.

### 2.3 Crop yield measurements

Grain and above-ground biomass yield measurements were estimated from 5 rows × 2 m yield plots in the center of each plot after physiological maturity. Cobs from the yield plots were shelled, maize grain weight and moisture content were immediately recorded. Sub-samples for stover and cores were taken and dried at 70 °C for moisture correction. Maize grain yield was calculated on a per hectare basis at 12.5 % moisture content and stover on dry weight basis.

### 2.4 Statistical analysis

This analysis focused on maize genotype performance and excluded cowpea due to the absence of a sole cowpea treatment. Maize grain yields were subjected to the Shapiro-Wilk normality test (Shapiro and Wilk, 1965). The data did not satisfy the assumption of normality and were thus log-transformed before analysis. The log-transformed data exhibited homogenous variance (*p* < 0.05) as confirmed by the Bartlett’s test (Snedecor and Cochran, 1989). The generalized linear model (GLM) was fitted by REML option using the R-package *ade4* in R-Studio Version 0.99.892 (Rstudio, 2016)

to estimate the effects with interaction of tillage method, cropping system and maize genotype on maize grain yield. Initially data was analyzed by individual season to assess the performance of the tested factors. In the overall analysis, genotype, tillage and cropping system were considered fixed factors whereas cropping season nested within year, and replication were considered random factors. All statistical analyses were done in R-Studio Version 0.99.892 (Rstudio, 2016)

and all the graphs were plotted in RStudio using the package *ggplot2* (Wickham, 2009).

Yield penalty (YP) of intercropping was calculated as the percentage difference between yield in intercrop and corresponding sole crop for each genotype and tillage treatment. Yield penalty was calculated as (Eqn. 1):

(1) $$\text{Y}\text{P}\text{\%}=\left(\frac{{\bar{X}}_{i}-{\bar{X}}_{s}}{{\bar{X}}_{s}}\right)\times 100$$

where ${\bar{X}}_{i}$ is the yield the intercropping treatment (genotype and tillage technology), and ${\bar{X}}_{s}$ is the yield of the sole cropping treatment (control group). In intercropping, the main crop needs to be maintained such that the yield of the companion crop becomes an additional benefit. A lower (absolute value) or no yield penalty, meaning maintenance of the main crop, is desirable

A Z-score was calculated for each genotype to assess how the overall performance deviated from the mean (Eqn. 2):

(2) Z=(X−μ)/σ

where *Z* is the z-score, *X* is the mean yield of each genotype over the experimental period, *μ* is the combined mean for all the genotypes the experimental period, and *σ* is the standard deviation.

Yield stability of the maize genotypes was assessed through Additive Main Effects and Multiplicative Interaction Model (AMMI) analysis (Sabaghnia et al., 2008). The AMMI analysis first fits additive effects for genotypes and environments using the additive ANOVA procedure and then fits multiplicative effects for G × E (genotype × environment) by principal component analysis (PCA). An environment was defined as a combination of tillage, cropping and season nested within a year giving a total of 24 environments. The AMMI stability value (ASV) was calculated using the following formula (Eqn. 3), as suggested by Purchase (1997).

(3) $$ASV=\sqrt{\;\left(\left(\frac{{SS}_{PC1}}{{SS}_{PC2}}\right)\;\times {\;PC1}^{2}\right)+\;{PC2}^{2}}$$

where ASV is AMMI’s stability value, SS is sum of squares, PC1 is interaction of PC one, PC2 is interaction of PC two. The higher the PCA score, either negative or positive, the more specifically adapted a genotype is to a certain environment. The genotypes with the lowest ASV values are considered to be the most stable.

Rainfall variability was calculated as the normalized anomaly using the following formula:

(4) $$N=(X-\;\bar{x)}\;\;/\text{σ}$$

where X is the amount of rainfall per season, $\bar{x}$ is long-term seasonal average rainfall, and σ is the standard deviation of rainfall for the same period.

## 3 Results

### 3.1 Crop productivity

Individual season analysis of data revealed that in the short rainy season of 2013 and long rainy season of 2016, tillage and cropping had a significant effect on maize yield (p < 0.05). In the long rainy season of 2014 and short rainy season of 2015, genotype and cropping had a significant effect on crop yield whereas in the short rainy season of 2014, genotype and tillage were significant. In the long rainy season of 2015, only tillage had a significant effect on crop yield. All the three factors tested had a significant effect in four of the six seasons. Overall, maize genotype (p < 0.001) and cropping systems (p = 0.02) had a significant effect on maize grain yield, but tillage (p = 0.303) did not (Table 1). However, the interactions of these factors did not have a significant effect on maize yield. The largest maize grain yield of 7.94 t ha^−1^ was recorded for the genotype CKH10085 in sole cropping under CT (Table 2). The same genotype recorded the highest yield of 7.02 t ha^−1^ under intercropping also under conventional tillage. In the no-till treatment, genotype CKH10717 recorded the highest yields of 7.49 t ha^−1^ and 6.62 t ha^−1^ in sole and intercropping treatments, respectively. Genotypes that performed well under CT did not necessarily yield as high under no-till conditions, and this pattern was consistent under both sole and intercropping. For example, genotype H513 recorded 7.7 t ha^−1^ under CT, but recorded only 6.91 t ha^−1^ under no-till. Genotype CKH10717 was the only genotype to perform better under no-till compared with CT – the recorded yield under CT was 7.27 t ha^−1^ and 7.49 t ha^−1^ under no-till (Table 2).

**Table 1:** Summary of the output of the generalized linear mixed model (GLMM) showing the effect of maize genotype, cropping and tillage systems on maize grain yields over six cropping seasons (2013–2016) in on-station trials at Kiboko, Kenya.

Source	*DF*	*F-Value*	*P-Value*
*Cropping*	1	47.82	0.020
*Tillage*	1	1.88	0.303
*Genotype*	5	9.95	0.001
*Year*	3	15.51	0.061
*Season (Year)*	2	0.93	0.526
*Cropping × Tillage*	1	0.05	0.837
*Cropping × Genotype*	5	0.94	0.494
*Tillage × Genotype*	5	0.89	0.521
*Genotype × Season(Year)*	10	0.64	0.751
*Cropping × Season(Year)*	2	0.87	0.525
*Tillage × Season(Year)*	2	8.68	0.062
*Cropping × Tillage × Genotype*	5	2.68	0.087
*Cropping × Tillage × Season(Year)*	2	1.78	0.218
*Tillage × Genotype × Season(Year)*	10	1.55	0.251
*Cropping × Genotype × Season(Year)*	10	1.28	0.352
*Cropping × Tillage × Genotype × Season(Year)*	10	0.5	0.890

**Table 2:** The effect of maize genotype, cropping and tillage systems on maize grain yields over six cropping seasons (2013–2016) in on-station trials at Kiboko, Kenya.

Cropping season	Maize genotype	Conventional tillage (t ha^−1^)		Conservation tillage (t ha^−1^)	
		Sole crop	Intercrop	Sole crop	Intercrop
2013B	CKH08051	5.5	5.7	7.7	6.2
	CKH10077	7.1	4.5	5.4	6.5
	CKH10080	7.3	4.7	7.3	7.1
	CKH10085	6.4	4.5	8.7	6.3
	CKH10717	5.8	5.3	7.8	7.6
	H513	8.6	6.1	8.0	6.5
2014A	CKH08051	8.5	8.0	7.7	7.7
	CKH10077	7.6	6.8	7.1	6.6
	CKH10080	8.1	6.8	7.7	7.8
	CKH10085	8.4	7.9	8.1	8.1
	CKH10717	8.0	8.0	8.3	8.5
	H513	7.6	6.9	8.0	7.8
2014B	CKH08051	9.0	9.0	8.6	7.4
	CKH10077	8.1	8.7	7.2	7.1
	CKH10080	9.2	8.5	8.1	8.4
	CKH10085	10.0	9.9	8.6	8.7
	CKH10717	9.3	8.9	9.4	7.9
	H513	9.0	8.9	7.9	6.9
2015A	CKH08051	5.5	6.5	4.9	4.8
	CKH10077	6.2	5.6	4.8	4.6
	CKH10080	6.3	6.4	4.4	4.8
	CKH10085	7.0	7.3	4.7	3.1
	CKH10717	6.4	6.8	6.0	3.5
	H513	6.2	5.9	4.6	5.7
2015B	CKH08051	5.3	5.9	5.1	3.8
	CKH10077	4.4	4.8	5.0	3.7
	CKH10080	5.6	4.5	5.2	4.2
	CKH10085	6.7	5.7	6.3	5.1
	CKH10717	5.4	5.3	5.8	5.5
	H513	6.3	5.1	5.9	5.3
2016A	CKH08051	8.6	6.3	7.0	5.9
	CKH10077	8.2	6.5	7.0	6.1
	CKH10080	8.6	7.3	7.1	5.5
	CKH10085	9.2	6.9	8.1	6.1
	CKH10717	8.7	7.3	7.7	6.7
	H513	8.5	6.4	7.0	5.4
*Standard error*		0.16	0.16	0.15	0.17

**Fig. 1:**
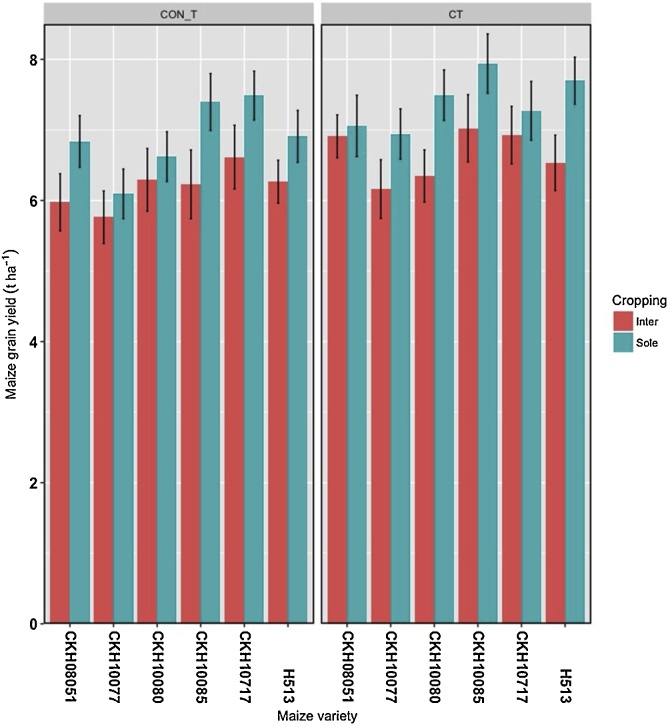
Productivity of six genotypes in sole and intercropping with cowpea and under conventional (CT) and conservation tillage (CON_T) practices for six cropping seasons (2013–2016) in on-station trials at Kiboko, Kenya.

**Fig. 2:**
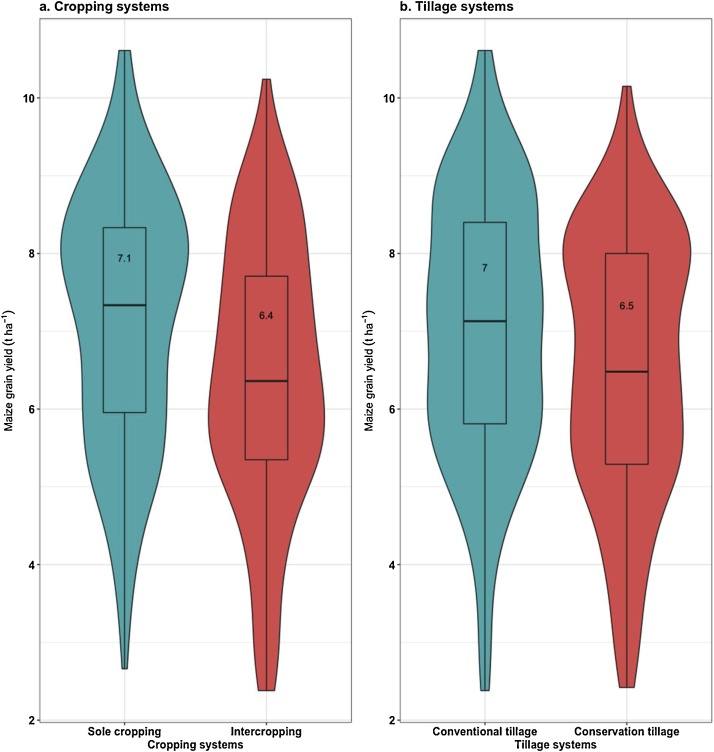
Combined violin and boxplot to show the overall distribution of maize grain yield in (a) two cropping systems, and (b) two tillage systems over six seasons at Kiboko, Kenya.

**Fig. 3:**
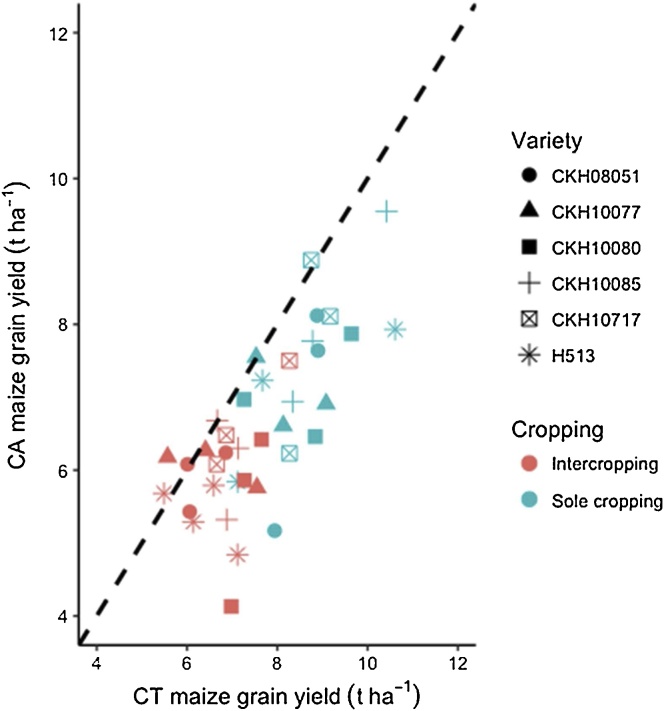
Relative comparison of maize grain yield in tillage and cropping systems treatments for the six genotypes tested over six seasons at Kiboko, Kenya.

Considering the effect of cropping and genotype, CKH10085 yielded highest (7.7 t ha^−1^) under sole cropping but had the joint (with H513) highest yield penalty due to intercropping of 1.1 t ha^−1^ (Fig. 1). The effect of intercropping on maize yield showed an overall (across genotypes and tillage) yield penalty of 0.7 t ha^−1^ (Fig. 2a). The potential beneficial effects of no-till were diminished compared with CT as the no-till plots consistently recorded lower yields (Fig. 2b, Fig. 3). Overall, no-till depressed yield compared with CT by about 0.5 t ha^-1^ (Fig. 2b). A relative comparison of maize productivity under no-till and CT also clearly showed that no-till depressed yields across maize genotypes and cropping systems (Fig. 3). When tillage system and genotype were considered, maize genotype CKH10085 still recorded the largest yield of 7.5 t ha^−1^ but under CT (Fig. 2).

**Fig. 4:**
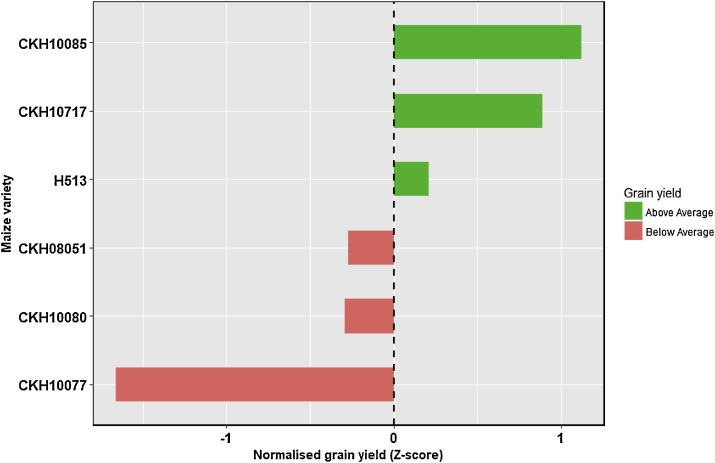
Diverging bar chart to show the variance of varietal yield above and below an average yield based on performance of the six genotypes tested over six seasons at Kiboko, Kenya.

The largest yield penalty of -17 % was recorded with the genotype CKH10085 under no-till, and a slight positive improvement was recorded with genotype CKH08051 under CT (Table 3). Results suggest that an intercropping yield penalty of 10 % is not significant. The *Z*-scores showed that genotype CKH10085 performed well above average with a score of 1.12, and CKH10077 was well below with a score of -1.66 (Fig. 3). The six genotypes were separated equally into two broad categories based on yield performance (Fig. 4).

**Table 3:** The overall effect of intercropping on maize grain yield penalty as affected by genotype and tillage systems over six cropping seasons (2013–2016) in on-station trials at Kiboko, Kenya.

Genotype	Conventional tillage (%)	Conservation tillage (%)
CKH08051	0.1	−12.8
CKH10077	−10.2	−5.2
CKH10080	−15.4	−5.1
CKH10085	−12.0	−17.4
CKH10717	−4.1	−12.7
H513	−14.7	−7.2
Mean	−9.4	−10.0
*Standard error*	5.6	5.8

### 3.2 Genotype stability analysis

Stability analysis results showed that the highest yielding genotypes were not necessarily the most stable. Genotype CKH10080 was the most stable with ASV value of 0.49 (Table 4). The highest yielding genotype (CKH10085) was only sixth in terms of stability. On the other hand, the most stable genotype was only fifth in terms of yield (Table 4). The commercial genotype H513 was significantly least stable than the other experimental genotypes with the highest stability value (ASV) of 1.97.

**Table 4:** Stability indices of the six maize genotypes based on grain yield over six cropping seasons (2013–2016) in on-station trials at Kiboko, Kenya.

Genotype	ASV	YSI	rASV	rYSI	Mean
CKH10080	0.49	6	1	5	6.67
CKH08051	0.61	6	2	4	6.69
CKH10717	1.35	9	3	2	7.07
CKH10077	1.40	6	4	6	6.24
CKH10085	1.78	6	5	1	7.15
H513	1.97	9	6	3	6.85

*ASV =AMMI stability value, *YSI* = Yield stability index, *rASV* = Rank of AMMI stability value, *rYSI* = Rank of yield stability index, *Mean* = average genotype by environment.

## 4 Discussion

### 4.1 Crop productivity

It was evident that intercropping and conservation tillage depressed maize grain yield. The intercropping option tested was an additive maize-cowpea intercropping system in which the plant population of maize (the main crop) was maintained in both sole and intercrops. The planting pattern had a maize row alternating with a cowpea row, and with spacing of 37.5 cm between rows of maize and cowpea. These narrow rows appeared to have caused significant interspecific competition among the component crops. The main benefits of intercropping are achieved when competition is reduced and complementarity and facilitation increased (Willey and Rao, 1980; Rusinamhodzi et al., 2012, 2017). The yield penalty of intercropping recorded in this study across all genotypes suggested that an alternative intercropping system could have led to better yields of the component crops. For example, the MBILI system (cf. Mucheru-Muna et al., 2009) where two maize rows alternate with two rows of legume has been found to reduce competition and ensure high yields of both companion crops and is more profitable. Similarly, Rusinamhodzi et al. (2012) reported that alternative planting arrangements may help to maintain the yield of the main crop as competition in cereal-legume intercrops in not necessarily for nutrients but light interception. In addition, poor productivity could have been caused by suboptimal NPK fertiliser formulations for the intercrops. We applied DAP (18 %N: 46 %P2O5: 0 %K) at planting and maize was top-dressed by urea. To the best of our knowledge, no suitable fertiliser formulations currently exist for cereal-legume systems in SSA, and this warrants further research.

The retention of crop residues in the no-till systems did not result in increased yields relative to the CT systems across the seasons. Two scenarios could explain this observation. Firstly, the rate of fertiliser application was the same across the tillage systems, which meant that the crop residues could have immobilised some of applied N resulting in the crop accessing less N in no-till compared with CT plots. The immobilization of N in no-till plots has been reported previously (e.g. Masvaya et al., 2017) due to the wide C/N ratio of maize stover (Cadisch and Giller, 1997). Secondly, by the nature of their function, crop residues could have retained more water in the no-till plots compared with CT resulting in waterlogging (Araya and Stroosnijder, 2010), reduced aeration and further nutrient unavailability for crop uptake (Cannell et al., 1985; Cannell and Hawes, 1994). Additionally, crop residue mulch tended to harbour several insects (data not presented), and this affected to a large extent the establishment of the crop in the no-till plots. Conservation agriculture plots have often been associated with increased biodiversity, but also pests and disease build-up which result in lower yields or alternatively increased costs of control (e.g. Nawaz and Ahmad, 2015; Brainard et al., 2016).

**Fig. 5:**
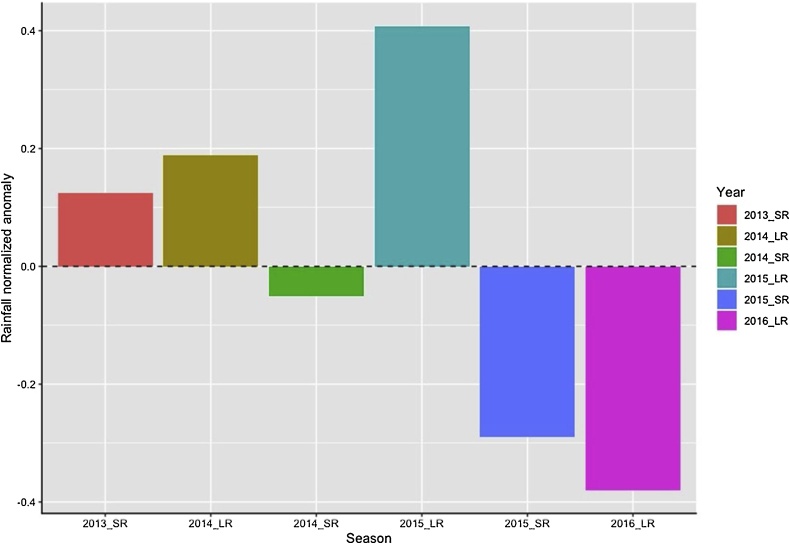
Variability of rainfall around the mean for the six cropping seasons at Kiboko Research Station, Kenya.

The cropping season did not exert a significant influence on grain yield because the variability of rainfall and its potential effect on crop production was minimized by supplemental irrigation. Rainfall variability for the experimental period was large with normalized anomaly values ranging from -30 % to 40 % (Fig.5), though the mean of 154 mm per season was too low to have a significant effect on crop productivity. The amounts of rainfall received underlines the risk associated with relying heavily on natural rainfall, and the decision to use supplemental irrigation also highlights the investments needed under rainfed conditions to reduce such risk. Supplemental irrigation allowed us to test the performance of elite maize genotypes under different cropping and tillage systems which was impossible when relying entirely on rainfall. The success of manipulating tillage to ameliorate rainfall shortage depends largely on the extent of the deficit to be covered, and may not be relevant for example to the climatic conditions of our study site. In literature, it has been observed that prolonged dry periods may cause the benefits of mulching to diminish due to continued evaporation (e.g. Jalota and Prihar, 1990). Results suggested that sustainable intensification may be possible under rain-fed conditions if the climatic risk related to rainfall can be reduced. For poorly resourced households who constitute the majority, effective low-cost but labour intensive options such as tied-ridges (Araya and Stroosnijder, 2010) and the *Zai* system (Roose et al., 1999) can be considered. The *Zai* pits and tied-ridges work in the same way by capturing rainfall and run-off, allowing water to infiltrate into the soil profile. When these options are combined with mulch cover as in our study, evaporation from the soil surface is reduced resulting in additional moisture retention and use (Serraj and Siddique, 2012). Although our study showed the importance of supplemental irrigation to reduce the risk of rainfall variability, only a few farmers can invest in this type of infrastructure at present. In addition, weak water governance institutions and poor market integration prohibit the majority of smallholder farmers from profitable investments in irrigation (Mwamakamba et al., 2017).

The differences in performance of maize genotypes under both sole and intercropping suggested the need to identify genotypes to be deployed according to the adaptive management needs. Adaptive management refers in this case to the choice of genotype, intercropping and or no-till. As stable genotypes are not necessarily the highest yielding ones, adaptive management is needed to find a compromise between risk reduction and yield maximization depending on the farmers’ circumstances. For example, genotype CKH10085 had the highest yield under sole cropping but it recorded the largest yield penalty under intercropping. On the other hand, genotype CKH10717 maintained the same average yield in both CT and no-till systems. This genotype by environment interaction has also been reported for example in Nepal where four maize genotypes performed significantly different when tested under a combination of tillage and residue management (Bk and Shrestha, 2014). The maize genotypes used in this study like many others are often bred under sole cropping, conventional tillage, and weed free and mostly adequate nutrient inputs. Such conditions at experimental stations are often different to the reality of farmers’ fields and the management they can afford. An ideal genotype should produce high yields regardless of environmental conditions (Zhang et al., 2015). This characteristic is important to cushion farmers against unpredictable rainfall coupled with their low and dwindling investment capacity.

### 4.2 Genotype and cropping systems stability

The genotypes CKH10080 and CKH08051 were more stable than the other experimental genotypes under the variable growing and management conditions in this study. These two genotypes are of intermediate maturity and drought tolerant, two critical attributes to increased maize production. This result suggested these two genotypes have the ability to achieve high potential yield when conditions are favourable – but more importantly achieve minimum yield reduction under unfavourable conditions (Martynov, 1990). However, the provision of irrigation water to supplement the inadequate rainfall received across seasons could have limited some of the genotypes to express their potential such as the ability to withstand prolonged dry periods. The hypothesis that some genotypes are more adaptive to biophysical and management changes was supported by the results. There is opportunity to deploy targeted genotypes to the local farmer practices to either reduce risk of crop loss with more stable genotypes such as CKH10080 or maximize yield in higher potential agro-ecologies with genotypes such as CKH10085.

### 4.3 Implications for sustainable intensification

Sustainable intensification is underpinned by developing appropriate agronomic practices including adaptive nutrient management (cf. Roberts, 2007), crop combinations and sequences, and using genotypes that are adapted to the environmental conditions that prevail in the target areas, and the market demand (e.g. Sanginga and Woomer, 2009). Farmer management decisions such as genotype selection, planting date, and plant density can affect the yield potential at a given site by influencing the utilization of available solar radiation, soil moisture reserves and nutrients during the growing season (Evans and Fischer, 1999). If the real conditions of farmers are to be considered i.e. inadequate fertiliser application and inability to access and use appropriate herbicides (Baudron et al., 2012), it means the yields reported here would have been much smaller. Similarly, genotypes should not be promoted based on performance under sole cropping with good nutrition and other management but after vigorous testing under conditions similar to the management in farmers’ fields (Spiertz, 2013).

The overall effect of intercropping was shown in this study to have an average yield penalty of 0.7 t ha^−1^ on maize productivity. This study solely focused on maize productivity only, and the critical question is whether intercropping is the best cropping system and whether the companion legume yield is large enough to offset the loss in maize gain yield. The solution depends on the production objective of the farmer, and how the different crops are used at farm level. Intercropping is likely to be more suitable in situations of land constraints and high land utilization where crop rotations may not be feasible (Rusinamhodzi et al., 2012). The right choices for farmers to utilize these new technologies depend heavily on information dissemination, in forms that are suitable to the end users (Sanginga and Woomer, 2009). The information given to stakeholders is important to raise awareness and involve them in technology development in a participatory manner (Chiffoleau and Desclaux, 2006). Participatory development and testing of new technologies is essential (Cerf et al., 2012), considering the conditions (biophysical and cropping systems) where the crop genotypes will be used, as well as the essential inputs of the ultimate end users.

Yield stability analysis for the six maize genotypes suggested tradeoffs between yield maximization and risk reduction for farmers. The unpredictable nature of rainfall and its distribution across seasons coupled with risk-averse (Chibnik, 1981) nature of farmers may suggest that they are likely to choose stability over yield maximization. Improved nutrient management improves yields substantially but can also increase yield variability across seasons (Kihara et al., 2016). Similarly, while improved maize genotypes may increase yields, results reported here also indicate that yields can be more variable. Thus, improved genotypes should be deployed in combination with careful consideration of the farmer’s circumstances.

## 5 Conclusion

Crop production in the semi-arid regions of Kenya is constrained by inadequate and often erratic rainfall exacerbated by low soil fertility. The study sought to understand how maize genotypes can be combined with adaptive management strategies such as intercropping and reduced tillage to achieve sustainable intensification under these conditions. Results showed that maize genotypes performed differently due to differences in cropping system but not necessarily tillage management. The differences in performance of genotypes revealed opportunities to deploy genotypes in response to the needs such as risk reduction or yield maximization – depending on the biophysical circumstances and production objective. Intermediate maturity maize genotypes with drought tolerant traits are needed to achieve sustainable intensification under water limited conditions. Intercropping maize and legumes may reduce maize yields, and better planting designs and custom blended fertilizers suitable for intercrops are needed to maintain the density of the main crop, reduce competition, and nourish both companion crops for improved productivity.

## CRediT authorship contribution statement

**Leonard Rusinamhodzi:** Formal analysis, Visualization, Writing - original draft, Writing - review & editing, Supervision. **Dan Makumbi:** Conceptualization, Methodology, Writing - review & editing. **James M. Njeru:** Investigation, Data curation. **Fred Kanampiu:** Conceptualization, Methodology.

## Declaration of Competing Interest

The authors declare that they have no known competing financial interests or personal relationships that could have appeared to influence the work reported in this paper.
